# Remediation of antimony-contaminated soil using food waste organic fertilizer and rhizosphere microbial response mechanism

**DOI:** 10.3389/fmicb.2025.1521692

**Published:** 2025-02-26

**Authors:** Shenglian Luo, Yingxuan Ouyang, Weimin Zeng, Xiaoyan Wu

**Affiliations:** ^1^School of Resources Environment and Safety Engineering, University of South China, Hengyang, China; ^2^School of Minerals Processing and Bioengineering, Central South University, Changsha, China

**Keywords:** food waste organic fertilizer, antimony-contaminated soil remediation, *Pteris vittate*, antimony oxidation, microbial community

## Abstract

Antimony pollution poses a significant threat to both the ecological environment and the health of people living in mining regions. Using organic fertilizers is an efficient approach for the remediation of heavy metal contamination in soil. This study aimed to explore how food waste organic fertilizer (FF) can remediate antimony-contaminated soil and the associated rhizosphere microbial response mechanism. The analysis of soil physicochemical properties revealed that the application of FF notably reduced bulk density (from 1.57 to 1.08 g cm^−3^), enhanced salinization levels, and increased the content of organic matter, available nitrogen, phosphorus, and potassium (*p* < 0.05). In the FF group, the plant height of *Pteris vittate* increased by 82.12% compared to the control group. The antimony valence state analysis revealed that after applying FF, the Sb(III) content in the rhizosphere and endosphere of *P. vittate* was significantly lower than that in other groups (*p* < 0.05), while the Sb(V) content in the endosphere was the highest. This indicated that FF can enhance the oxidation and detoxification of Sb(III) in the soil to produce Sb(V), which is then accumulated in the root of *P. vittate*. Microbial community analysis showed that the application of FF promoted the continuous enrichment of *Proteobacteria*, *Actinobacteria*, *Firmicutes*, and *Bacteroidetes* in the roots of *P. vittate*; this is particularly evident in the specific microbial groups with Sb(III) oxidation, nitrogen fixation, and phosphorus and potassium solubilization functions, including *Acinetobacter*, *Sphingomonas*, *Comamonas*, *Bradyrhizobium*, *Alphaproteobacteria*, *Acidovorax*, and *Paenibacillaceae*. These microbes help mitigate the adverse effects of poor soil conditions and heavy metals on the growth of *P. vittate* in mines. This study provides a new approach to resource utilization of food waste and the remediation of antimony-contaminated sites.

## Introduction

Hunan Province is rich in antimony (Sb) ore resources, with reserves ranking first in the country and accounting for over 20%. For a long time, there has been a serious phenomenon of private mining and disorderly excavation in antimony mining in many regions, leading to the loss of resources and also triggering the historical legacy of environmental problems. With a history spanning more than a century, the antimony contamination issue in Hunan Province poses a significant threat to soil biodiversity and the health of surrounding residents. Antimony’s toxicity varies with its form in the environment; for example, elemental antimony is more toxic than inorganic antimony salts, and inorganic antimony is more toxic than organic antimony (Yu et al., 2022). Although the most prevalent valence states of antimony are +5 and + 3, Sb(V) is significantly less toxic than Sb(III), being 10 times safer (Xiao et al., 2023). Previous research studies have reported that microorganisms such as *S. senarmontii* and *Bacillus* sp. are present. MLFW approach has the ability to oxidize antimony, transforming the highly toxic Sb(III) into much less toxic Sb(V) (Nguyen and Lee, 2014; Zhu et al., 2017). This approach represents an efficient and environmentally friendly method for remediation of antimony pollution. In addition, *P. vittate* is recognized as an arsenic hyperaccumulating plant, predominantly found in subtropical and tropical areas south of the Qinling Mountains, and has been widely used for phytoremediation of heavy metal pollution (Chen et al., 2005). Microorganisms can promote plant growth by increasing plant tolerance to stress and attenuating the biotoxicity of heavy metals in rhizosphere soil (Yu et al., 2021). Therefore, combined microbial-plant remediation of heavy metal pollution has increasingly attracted the attention of ecologists domestically and internationally.

Aerobic composting mature products are usually used as organic fertilizers, providing plants with the nutrients and trace elements needed for their growth and ultimately improving soil fertility and crop yields. Jia et al. (2019) found that the application of food waste organic fertilizer (FF) to orchard soil significantly improved the total organic carbon content and organic carbon distribution in the soil. Long-term application of food waste organic fertilizer was an effective measure for enhancing soil fertility and carbon sink capacity. Organic fertilizer made from food waste composting also has a regulating effect on soil bulk density, acidity, and alkalinity. In addition, the humic substances rich in compost products can act as electron shuttles for metal oxidation and reduction, complexing heavy metal ions. In particular, humic acid, which contains a variety of active functional groups, is an effective component for the adsorption and complexation of heavy metals (Ma et al., 2024). Karer et al. (2015) have studied the remediation effect of biochar, inorganic, and compost-based amendments on soil contaminated by heavy metals, and the results show that adding organic fertilizers can significantly reduce the content of extractable heavy metals in the soil. However, no reports on the remediation of antimony-contaminated soil by the application of organic fertilizers made from food waste are available in the literature.

In the previous study, we established an enhanced process for aerobic composting of food waste based on the inoculation of thermotolerant lignin-degrading bacteria and obtained mature organic fertilizer. The content of humic substance and total potassium, nitrogen, and phosphorus (NPK) reached 74.28 and 4.4%, respectively (Wu et al., 2022a). Furthermore, the mature materials also included various active microorganisms, particularly antimony-reducing and oxidizing functional microbiota, such as *Bacillus*, *Acinetobacter*, *Pseudomonas*, *Rhodospirillaceae*, *Enterobacter*, and *Sphingobaterium* (Wu et al., 2022b). Li et al. (2018) revealed that *Bacillus* sp. S3, isolated from the soil of arsenic antimony-contaminated mining areas, can oxidize the highly toxic Sb(III) to Sb(V); this process reduces the stress imposed by Sb(III) on *Arabidopsis thaliana* (L.) Heynh in the soil. Yang et al. (2015) discovered that *Pseudomonas* exhibits highly effective oxidation of As(III), converting it entirely to As(V) within a 20-h period, thereby effectuating the biological detoxification of arsenic. At the genus level, *Pseudomonas* and *Acinetobacter* represent 31.96 and 9.24% of the known Sb(III) oxidizing bacteria, respectively (Deng et al., 2021). The microbiome constitutes a vital biological component of soil ecosystems. Its abundance, composition, and activity significantly influence the sustainable productivity of the soil (Castellano-Hinojosa et al., 2023). Additionally, microorganisms exhibit a greater sensitivity to heavy metal pollution in soil compared to both animals and plants. The aforementioned properties of food waste-based organic fertilizer suggest that its application to antimony-contaminated soil can theoretically have a positive effect on improving soil structure, organic matter content, and microbial activity. However, it is unknown whether organic fertilizer made from food waste can promote antimony oxidation and detoxification, as well as its remediation effects and ecological mechanisms, which are worth studying.

Thus, this research primarily explores the effects of FF, derived from inoculating thermotolerant lignin-degrading bacteria, on remediating antimony-contaminated soil through field experiments. With no fertilization serving as the control, two additional groups using sheep manure organic fertilizer (SF) and inorganic fertilizer (IF) were established. The differences in soil physicochemical properties, growth of *P. vittate*, heavy metal content, and antimony valence transformation across various groups at different fertilization periods were investigated. Utilizing high-throughput sequencing technology, microbial community analysis was performed on the rhizosphere soil samples of *P. vittate* to understand the microbial response mechanism behind the remediation of antimony-contaminated soil using FF.

## Materials and methods

### Experimental design

The field trial was conducted from August 2022 to October 2022 in the historical legacy mining area of Hehuang Village, Dafu Town, Anhua County, Hunan Province (28°17′42″N, 111°55′53″E), which has a long history of antimony mining resources. The test area has a subtropical monsoon climate, with an altitude of 216.6 m above sea level, an average annual temperature of 16.2°C, a yearly rainfall of 1,687 mm, a yearly sunshine hour of 1,426 h, and an annual frost-free period of 295 days. The test soil was a chalky clay loam. The surface soil (0–20 cm) had a pH of 6.78 and EC of 127.1 μS cm^−1^. It contained 70.54 g kg^−1^ organic matter (OM), 29% moisture content (MC), 1.14 g kg^−1^ total nitrogen (TN), 183.33 mg kg^−1^ available nitrogen (AN), 128.23 mg kg^−1^ available phosphorus (AP), and 72.29 mg kg^−1^ available potassium (AK). FF was the mature product obtained by aerobic composting of food waste and sawdust in a ratio of 3:1 (w/w) for 42 days (Wu et al., 2022b), followed by air drying and fine grinding before passing through a 2-mm nylon sieve. SF and IF were purchased from Anhui Fumin Biological Organic Fertilizer Co., Ltd. (Anhui Province, China) and Anyang Zhongying Fertilizer Co., Ltd., (Henan Province, China) respectively. The test plant was *P. vittate*, purchased from Jufeng Seed Industry Group Co., Ltd. (Anhui Province, China). The basic physicochemical properties of each fertilizer are shown in Table 1.

**Table 1:** Basic physicochemical properties of various fertilizers used in the study.

Fertilizer	OM (%)	MC (%)	TN (%)	AN (g kg^−1^)	AP (g kg^−1^)	AK (g kg^−1^)
FF	76.61	34.39	2.07	0.78	0.68	0.42
SF	43.8	24.5	2	0.66	0.62	0.49
IF	0	0.1	46	343.2	0	0

OM, organic matter; MC, moisture content; TN, total nitrogen; AN, available nitrogen; AP, available phosphorus; AK, available potassium; FF, food waste organic fertilizer; SF, sheep manure organic fertilizer; IF, inorganic fertilizer.

**Figure 1:**
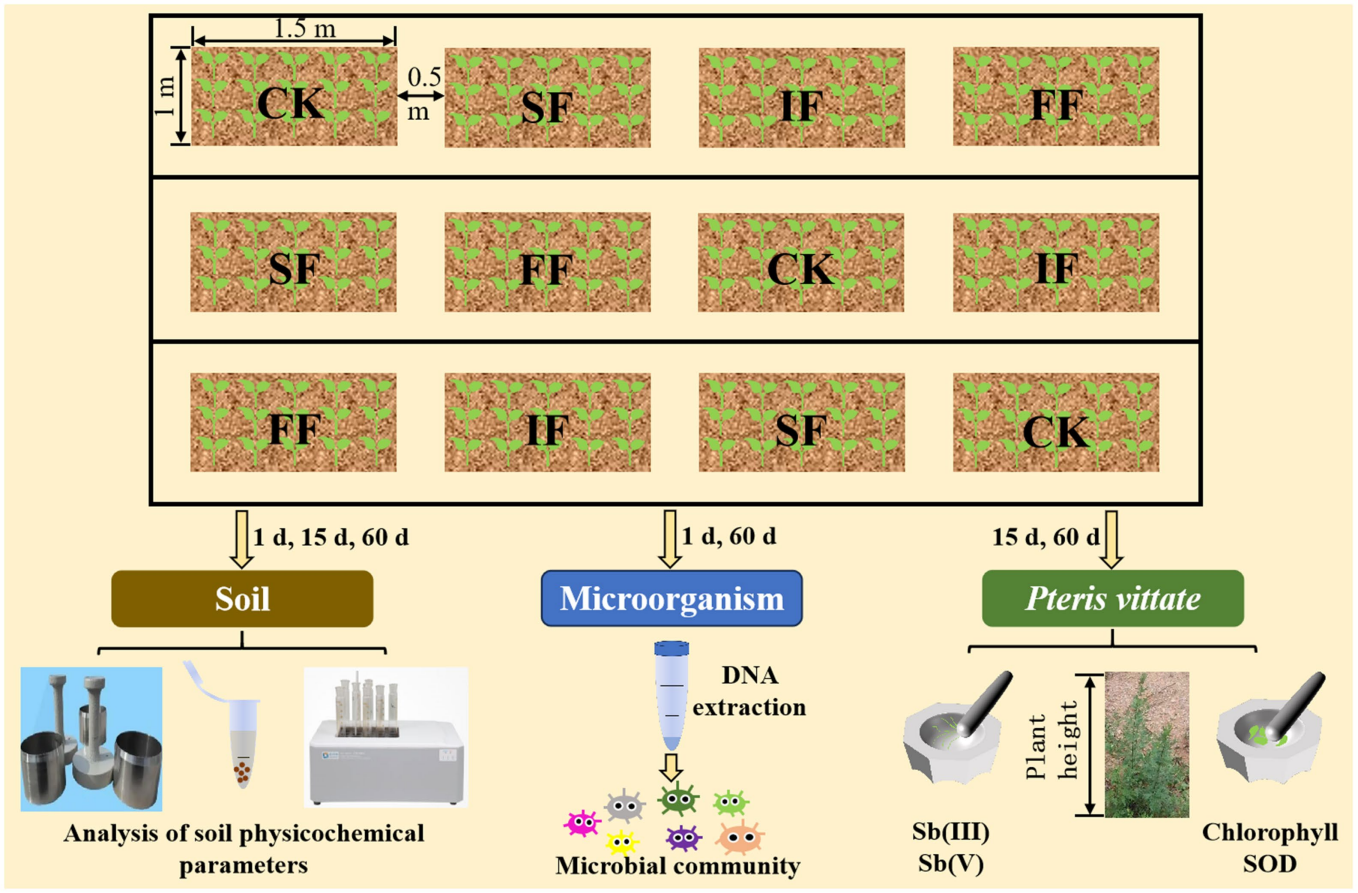
Experimental design diagram for food waste organic fertilizer remediation of antimony-contaminated soil. FF, food waste organic fertilizer; SF, sheep manure organic fertilizer; IF, inorganic fertilizer; SOD, superoxide dismutase.

Three treatment (FF, SF, and IF) groups and a control (CK) group were set up in this experiment and replicated 3 times, resulting in 12 plots. Among these four treatment groups, the three groups, excluding the control group, were fertilized with equal nitrogen at a rate of 300 kg hm^−2^. Each treatment had a plot area of 1.5 m^2^ (1.5 m × 1 m), and the plots were randomized in a group design, with an interval of 0.5 m between plots separated by a 60-cm-deep plastic film (Figure 1). Organic fertilizers were applied in the form of basal fertilizers during plowing, and no further topdressing was required at the later stage. Half of the IF was applied before transplanting *P. vittate*, and the top coat of the other half during the growth period. Seeds of *P. vittate* were sown beforehand in an open space near the experimental field and irrigated regularly. Seedlings (10–15 cm) of *P. vittate* with similar growth were selected and transplanted with soil to 12 experimental plots with about 20 g of soil per plant. *P. vittate* was planted in 15 plants (3 rows and 5 columns) in each experimental plot and irrigated at 5-day intervals.

### Soil and plant sampling method

About 100 g of soil samples were collected from the top layer of 0–20 cm by mixed sampling at multiple points on days 1, 15, and 60. Subsequently, the soil samples were air-dried, ground, and sieved through a 0.15-mm sieve to determine pH, EC, OM, AN, AP, AK, As, Hg, Pb, Cd, Cr, and Sb. In addition, 5 g of rhizosphere soil sample was taken, and root fragments and impurities were removed. After air drying and grinding, it was sieved through a 0.15 mm sieve and used to determine Sb(III) and Sb(V). After 2 months of growth, the *P. vittate* was used to collect plant samples. 10 g of root and leaf tissues were taken and placed in an oven at 105°C for 30 min, then dried at 60°C until constant weight, ground into a fine powder with liquid nitrogen, and passed through a 0.15-mm sieve. Plant height, chlorophyll, superoxide dismutase activity (SOD), and the content of Sb(III) and Sb(V) in roots were determined.

### Physicochemical analyses

Soil bulk density was determined using the ring knife method. The contents of AN, AP, and AK were determined using the alkaline diffusion method, hydrochloric acid-ammonium fluoride (HCL-NH_4_F) method, and ammonium acetate (NH_4_OAc) extraction flame photometer, respectively (Khan et al., 2021). Notably, 0.2 g of air-dried soil sample was placed in a digestion tube, and 5 mL of HNO_3_, 5 mL of HF, and 2 mL of HClO_4_ were added sequentially, shaken well, and left overnight. The digestion tube was heated on a graphite ablator for 1 h until the solution was clear. The concentration of As, Hg, Pb, Cd, and Cr in the solution was thereafter measured using an inductively coupled plasma emission spectrometer (ICP7400, ThermoFisher, USA). 2 g of air-dried soil samples were mixed with 15 mL of 0.1 mol L^−1^ citric acid (pH 2.0) and then placed in a water bath shaker at 70°C for 2 h at 180 rpm, followed by centrifugation at 3500 rpm for 30 min. The supernatant was filtered through a 0.45 μm membrane and divided into two fractions for the determination of Sb(III) and total antimony (Sb_total_) with the help of ICP7400, respectively (Xiao et al., 2024). Sb(V) content is the difference between Sb_total_ and Sb(III). OM, pH, and EC were determined as described by Wu et al. (2021).

The plant height was determined by using a soft ruler to measure the distance from the soil surface to the tallest point of the *P. vittate* leaves. Three leaves were randomly selected from each *P. vittate* plant to determine the plant’s chlorophyll content. The absorbance values of the samples at 645 nm and 663 nm were determined using the BC0990 Plant Chlorophyll Content Assay Kit (Solarbio, Beijing, China), which were the contents of chlorophyll a and chlorophyll b, respectively. The sum of the contents of chlorophyll a and chlorophyll b was the total chlorophyll content. The SOD activity of the plant root samples was determined by using the Nanjing Jiancheng Superoxide Dismutase (SOD) kit with a detection wavelength of 550 nm and zeroed in distilled water. The plant root tissues were dried in an oven at 105°C until constant weight, then ground to a fine powder by liquid nitrogen and passed through a 0.15 mm sieve. Immediately thereafter, antimony content in plant root tissues was determined according to the method for Sb(III) and Sb(V) determination in soil samples in the previous paragraph.

### DNA extraction and Illumina NovaSeq sequencing

Rhizosphere soil samples of *P. vittate* on day 1 and day 60 were collected for microbial community analysis. 5 g of soil samples were collected from each plot, root fragments, and large particulate matter were removed, placed in 10 mL sterile centrifuge tubes rapidly frozen in liquid nitrogen, and brought back to the laboratory to be stored at −80°C for DNA extraction. Genomic DNA samples from the CK, FOF, SOF, and IF groups were extracted using the DNeasy PowerSoil kit (QIAGEN, Dusseldorf, Germany). After verification of eligibility by 1% agarose gel electrophoresis, PCR amplification of bacterial 16S ribosomal RNA (rRNA) gene fragment was carried out with the primer pairs of 341F/805R (5′-CTACGGGNGGCWGCAG-3′/5′GACTACHVGGGTATCTAATCC-3′). The amplification product was subsequently purified, recovered, and then sent to Lianchuan Biotechnology Co. Ltd. (Hangzhou, Zhejiang, China) for bacterial 16S sequencing using the Illumina NovaSeq PE250 platform (Zhejiang Province, China). All raw data from sequencing were uploaded to the National Center for Biotechnology Information Sequence Read Archive (NCBI SRA) database (SRP539288).

### Bioinformatic analysis and statistics

After splicing, quality control, and chimeric filtering of the raw sequenced reads using QIIME2, high-quality CleanData was obtained. Then, the DADA2 q2-plugin was applied to get the unique Amplicon Sequence Variants (ASV, based on the percentage of non-clustered unique sequences) feature table and feature sequence (Luo et al., 2022). Diversity analysis and taxonomic identification at the phylum and genus levels of microbial communities were accomplished based on the ASV feature table and feature sequence using the SILVA database. The *α*-diversity of the microbial community was characterized by Chao1 and Shannon indices with the help of the Phyloseq R package. The *β*-diversity was analyzed using principal coordinate analysis (PCoA) based on the Bray–Curtis distance metric to reveal the similarities and differences among different samples. Microsoft Excel 2016 and Origin 2021 were utilized to handle all experimental data, and the results were shown as mean ± standard error of the triplicate. The statistically significant level was considered at *p* < 0.05.

## Results and discussion

### Effects of FF on the physicochemical properties of antimony-contaminated soil

**Figure 2:**
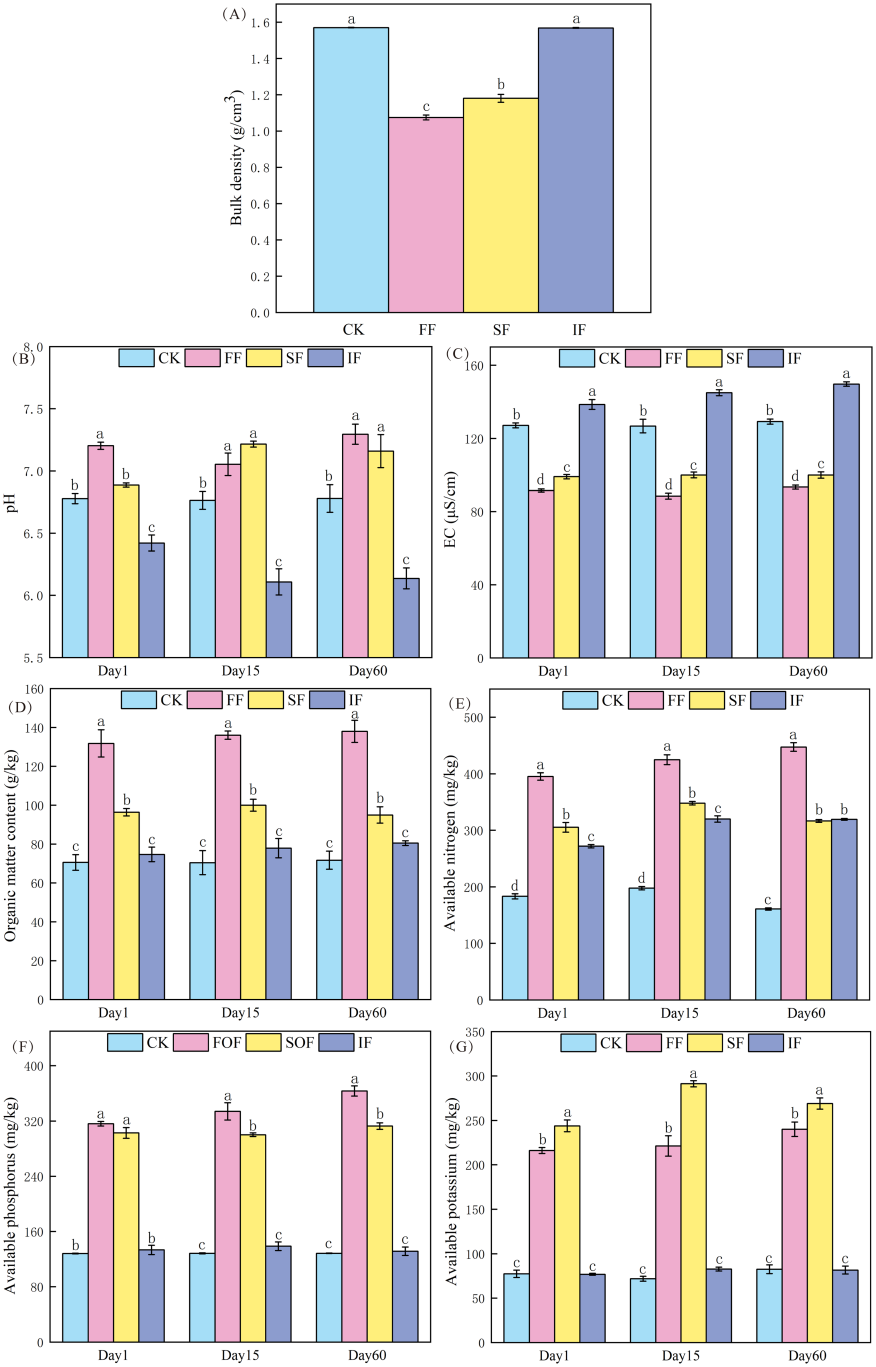
Effect of different fertilization treatments on **(A)** bulk density, **(B)** pH, **(C)** EC, **(D)** organic matter, **(E)** available nitrogen, **(F)** available phosphorus, and **(G)** available potassium of antimony-contaminated soil.

Soil bulk density is an essential indicator for measuring soil density, permeability, and air permeability (Walter et al., 2016). In general, reduced bulk densities are associated with looser soil structure, improved air permeability, and a more favorable environment for plant growth and development. As shown in Figure 2A, the top layer of antimony-contaminated soil showed the most significant (*p* < 0.05) reduction in bulk density from 1.57 to 1.08 g cm^−3^ after applying FF for 2 months, which was better than 1.18 g cm^−3^ in the SF group, and there was no significant difference in the IF group. In this study, the FF has a high porosity between particles and contains abundant active microorganisms and humic substances (72%). Applying it to antimony-contaminated soil can increase the total amount of microorganisms, and the activity of indigenous microorganisms, which produces more cracks and pore spaces, resulting in a reduction in the bulk density of the mine soil. As a fern plant, the growth of *P. vittate* requires soil rich in humic substance, loose, breathable, and fertile. The FF group’s lower bulk density suggested that its soil has the best air permeability and water retention capacity, which could result in more favorable environmental circumstances for the growth and development of *P. vittate*.

The pH of the soil has an impact on enzyme activity, microbial reproduction, and plant growth. The original pH value of the soil in the experimental site was 6.78, which belongs to neutral soil (Figure 2B). After the application of IF, the pH value significantly decreased and remained at approximately 6.11. This result was attributed to the fact that the N element in the IF primarily exists in the form of NH_4_^+^, which was oxidized into nitrate under the action of microorganisms, thus releasing H^+^ and leading to soil acidification, which was detrimental to plant growth. After applying two types of organic fertilizers, the pH value increased and remained stable at approximately 7.2. Of these, applying FF considerably raised the soil pH value (*p* < 0.05). In contrast to the findings of several previous researchers, Hong et al. (2017) observed that soil pH was decreasing rapidly, followed by a gradual recovery after applying various types of organic fertilizers. This may be due to the differences in the response of different soil types to applying different organic fertilizers. *P. vittate*, however, has calcium-loving characteristics and is mainly found in alkaline soils (Huang et al., 2024). Therefore, the soil treated with organic fertilizer in this study was more suitable for the growth and development of *P. vittate*.

It is generally accepted that an excess of 1,000 μS cm^−1^ in the soil EC value signifies an accumulation of surface salts and increased salinization, both of which are detrimental to the growth and development of plants (Tallarita et al., 2023). In this study, the original soil EC value was 127.1 μS cm^−1^ (Figure 2C). The IF application resulted in a considerable increase in EC value, reaching 149.73 μS cm^−1^ on the 60th day, which led to enhanced salinization of the surface soil, and may induce sluggish plant growth or even the phenomena known as “seedling burning.” Notably, organic fertilizers, particularly FF, could dramatically reduce the EC value, which kept the EC value in the range of 88.47–93.39 μS cm^−1^. This suggested that applying FF might effectively promote the desalination capacity of mine soil and speed up the release of late-acting salts, such as N, P, and K, in the surface soil.

Soil OM serves as a source of organic and inorganic nutrients for plants and an energy source for soil microbes. It is essential for developing the fundamental soil structure and a significant indication of soil fertility. The changes in OM content under various treatments are shown in Figure 2D. Throughout the study period, the OM content of the FF and SF increased by 86.83–93.15 and 32.41–36.6%, respectively, compared with the control group. The OM content increased slightly after applying IF. However, the difference was not significant (*p* > 0.05). The aforementioned results indicated that FF has the most important effect in increasing the OM content of mine soil, outperforming commercially available SF and IF. This relates to the fact that commercially available SF only contains 43.8% OM, and IF does not include OM, whereas FF contains 76.61% OM. Man et al. (2021) also found that the application of food waste compost products had a better effect on increasing soil OM content than chicken manure and pig manure organic fertilizers, with an increase of 11.64 and 19.87%, respectively. In addition, it can be observed from Figure 2B that the OM content in the same group did not show significant changes over time and remained stable.

Soil AN, AP, and AK reflect the soil’s fertilizer supply capacity of N, P, and K. They are exchangeable and water-soluble nutrients that can be exchanged or directly absorbed and utilized by plants from soil colloids. The initial contents of AN, AP, and AK in the control group soil were 183.33, 128.23, and 77.29 mg kg^−1^, respectively (Figures 2E–G). The application of IF only significantly increased the content of AN (*p* < 0.05) to 272 mg kg^−1^. The AN, AP, and AK content considerably rose on the day of applying FF and SF. Compared to the control group, the FF group grew by 115.64, 146.48, and 179.55%, whereas the SF group climbed by 66.55, 136.13, and 215.54%, respectively. FF has a substantially greater AN content than SF due to its higher OM content (76.61%). Active organic carbon can offer an adequate carbon source for soil microbes, thereby boosting the reproduction of nitrogen-fixing bacteria and lowering nitrogen loss. Overall, applying FF has the most significant effect on enhancing the amount of available nutrients in soil.

### Effects of FF on the growth of *Pteris vittate*

**Figure 3:**
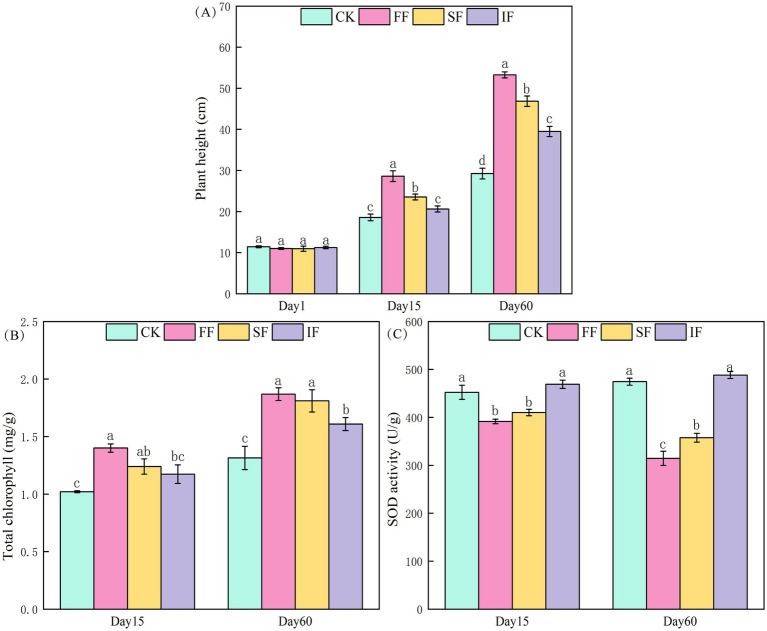
Effect of different fertilization treatments on **(A)** plant height, **(B)** total chlorophyll content, and **(C)** SOD activity of *Pteris vittate*.

A widespread antimony-rich plant in southwest China, *P. vittate* is a perennial herbaceous plant frequently used for the remediation of heavy metal-contaminated soil. To investigate the effect of FF on the growth of *P. vittate*, we measured the plant height, chlorophyll, and root SOD content of *P. vittate*. Plant height analysis showed that after 2 months of three fertilization treatments, the plant height of *P. vittate* was significantly increased compared to the control group (Figure 3A). With an increase of 82.12% over the control group, the FF group produced the most significant rise in plant height, followed by the SF group (60.23%) and the IF group (35.02%). The growth of *P. vittate* was roughly the same among the groups throughout the entire research period. According to the results of the chlorophyll measurement, there was no significant difference in chlorophyll content between the IF and control groups on the 15th day (*p* > 0.05). However, the organic fertilizer group’s chlorophyll content was significantly higher than the control group’s (Figure 3B). After 2 months, the chlorophyll content in the FF, SF, and IF groups was 1.87, 1.81, and 1.6 mg g^−1^, respectively, which were significantly higher than that of 1.31 mg g^−1^ in the control group (*p* < 0.05). The results of the SOD measurement showed that the SOD activity in the control group and IF group was significantly higher than that in the two organic fertilizer groups. On the 60th day, the SOD activity in the FF group was considerably lower than that in the SF group (Figure 3C).

The growth of *P. vittate* in antimony-contaminated soil was influenced by various factors such as soil structure, OM content, available NPK nutrient content, and heavy metal stress. In this study, the application of FF was found to be more effective than other treatments in reducing soil bulk density, alleviating soil acidity, reducing salinization, and enhancing the content of organic and inorganic nutrients. Consequently, *P. vittate* had the highest growth rate in this group. As the primary pigment for photosynthesis in plants, the fluctuation of chlorophyll content can directly reflect the growth and development of plants (Yu et al., 2024). The results of chlorophyll determination also reflect that the growth ability of *P. vittate* in the FF group was more potent than in other groups. When plants are exposed to heavy metals and other adversities, a considerable number of superoxide free radicals will be generated, resulting in the accumulation of reactive oxygen species (ROS), which can adversely affect or even destroy cell membranes, proteins, and DNA (Farooq et al., 2021). SOD is essential for scavenging the superoxide free radicals in plants, and its activity level reflects the degree of heavy metal stress in plants. The test soil was in a historical mining area and was contaminated with antimony. The SOD analysis results demonstrated that the *P. vittate* in the control group and IF group were most susceptible to heavy metal stress, followed by the SF group and the smallest in the FF group. Therefore, it can be inferred that applying FF can promote heavy metal detoxification and achieve remediation of antimony-contaminated soil. However, the content of heavy metals, antimony valence state transformation, and whether relevant functional microorganisms mediate them still remain unclear and require further research.

### Effects of FF on soil heavy metal content

The content of heavy metals in soil after 60 days of different fertilization treatments is shown in Table 2. There are significant differences in the content of As, Hg, Pb, Cd, Cr, and Sb, among which Sb has the highest content, ranging from 70.73 to 137.74 mg kg^−1^, and Hg has the lowest content, ranging from 0.069 to 0.078 mg kg^−1^. In prior research, we used *t*-tests to evaluate the background values and sample variations for each heavy metal. The findings indicated that, except for Sb, no substantial pollution was detected in the other five heavy metals (Wang et al., 2022). The application of IF considerably decreased the amount of Sb in the soil compared to the control group, but it had no discernible impact on As, Hg, Pb, Cd, or Cr. Following the application of IF, these heavy metals’ contents dropped; compared to the control group, Cr and Sb were significantly lower by 2.39 and 67.01%, respectively. After applying SF, the soil Sb content significantly dropped by 29.05% compared to the control group, while the Hg and Cd content did not change appreciably. More noteworthy is that the SF application significantly raised the content of As, Pb, and Cr by 4.73, 4.93, and 8.96 mg kg^−1^, respectively, compared to the control group.

**Table 2:** Heavy metal content in soil after 60 days of different fertilization treatments.

Treatment	Heavy metal content (mg kg^−1^)					
	As	Hg	Pb	Cd	Cr	Sb
CK	25.3 ± 2.17 ^b^	0.07 ± 0.00 ^a^	21.88 ± 0.53 ^b^	44.94 ± 2.93 ^a^	40.16 ± 1.10 ^b^	137.74 ± 3.46 ^a^
FF	22.34 ± 1.09 ^b^	0.069 ± 0.01 ^a^	20.05 ± 1.68 ^b^	42.08 ± 0.70 ^a^	37.77 ± 0.44 ^c^	70.73 ± 1.94 ^d^
SF	30.03 ± 0.96 ^a^	0.078 ± 0.01 ^a^	26.81 ± 0.97 ^a^	42.64 ± 0.68 ^a^	49.12 ± 0.79 ^a^	97.73 ± 2.17 ^c^
IF	23.86 ± 0.36 ^b^	0.069 ± 0.01 ^a^	21.01 ± 0.31 ^b^	45.51 ± 0.55 ^a^	38.9 ± 0.59 ^bc^	104.01 ± 0.50 ^b^

The superscript ‘abcd’ indicates significant differences.

The foregoing results indicated that both organic fertilizer treatments had a more significant effect on lowering antimony content in soil than the IF treatment, which was related to the abundance of humic substance in composting products. Humic substances can passivate heavy metals, thereby reducing the biological toxicity and mobility of heavy metals (Chen et al., 2024). However, the application of SF simultaneously increased the content of As, Pb, and Cr in the soil, which may be related to the fact that livestock and poultry breeding waste itself contains a certain amount of heavy metals (Guan et al., 2024). Pan et al. (2012) reported that long-term application of organic fertilizers from livestock and poultry in farmland soil can cause compound pollution of antibiotics and heavy metals, posing potential risks to the soil’s ecological environment. The usage of poultry manure boosted the risk of soil and vegetation pollution and harm to human health, following the study of Muhammad et al. (2020). Consequently, applying IF is an efficient and environmentally friendly way to remediate heavy metal pollution in mining soil.

### Effects of FF on the transformation of antimony valence state

**Figure 4:**
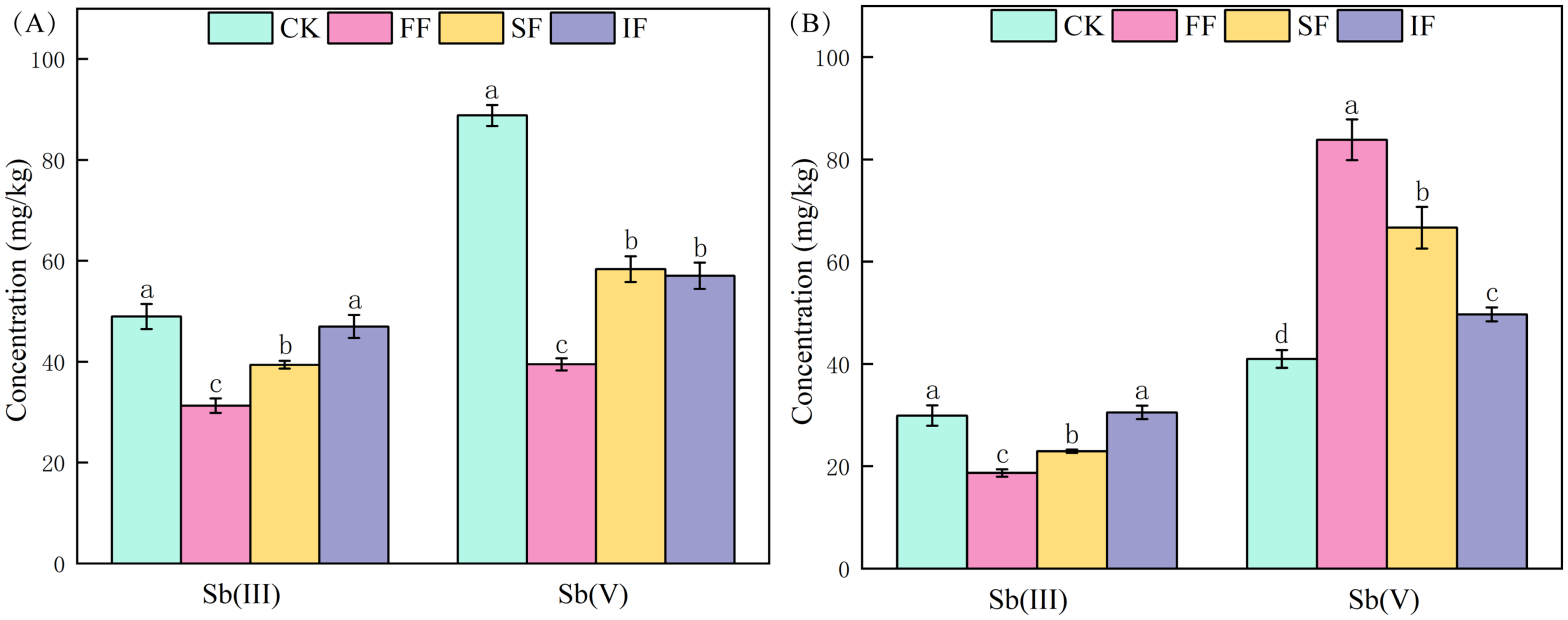
The content of Sb(III) and Sb(V) in **(A)** rhizosphere and **(B)** endosphere samples of *Pteris vittate* after 60 days of fertilization treatments.

To investigate the effect of FF on the transformation of antimony valence states in different ecological niches of *P. vittate*, we measured the Sb(III) and Sb(V) contents of rhizosphere and endosphere samples of *P. vittate* after 60 days of different fertilization treatments. The Sb(III) content in the rhizosphere samples was the lowest in the FF group (31.27 mg kg^−1^), followed by the SF group (39.39 mg kg^−1^), both of which were significantly lower than that in the IF and CK groups (*p* < 0.05). Meanwhile, the CK group’s rhizosphere sample had a considerably greater Sb(V) content than the other three fertilization groups, suggesting that more Sb was transferred to the endosphere of *P. vittate* in the three fertilization groups (Figure 4A). The determination results of antimony valence state content in endosphere samples of *P. vittate* showed that the Sb(III) content in the FF and SF was significantly lower than that in the IF and control groups (*p* < 0.05). In contrast, the Sb(V) content was significantly higher than that in the control group (Figure 4B).

Sb(III) is highly toxic, which is 10 times more toxic than Sb(V). Furthermore, the migration rate of Sb(III) in the environment is lower than that of Sb(V) (Herath et al., 2017). Following FF application, the Sb(III) contents in the rhizosphere and endosphere of *P. vittate* were significantly lower than that in other groups (*p* < 0.05), while the Sb(V) content in the endosphere was the highest, indicating that FF could promote Sb(III) oxidation and detoxification in polluted soil. In this group, *P. vittate* was the least exposed to Sb(III) toxicity stress, and its growth pressure was relieved. The results of the plant height and SOD activity measurements of *P. vittate* also supported this. It is speculated that the application of FF led to an increase in specific functional microbial taxa in the rhizosphere soil of *P. vittate*, promoting the oxidation of Sb(III) to Sb(V), which was then enriched by *P. vittate* and released into the endosphere.

### Rhizosphere microbial response mechanism for the remediation of antimony-contaminated soil by FF

**Figure 5:**
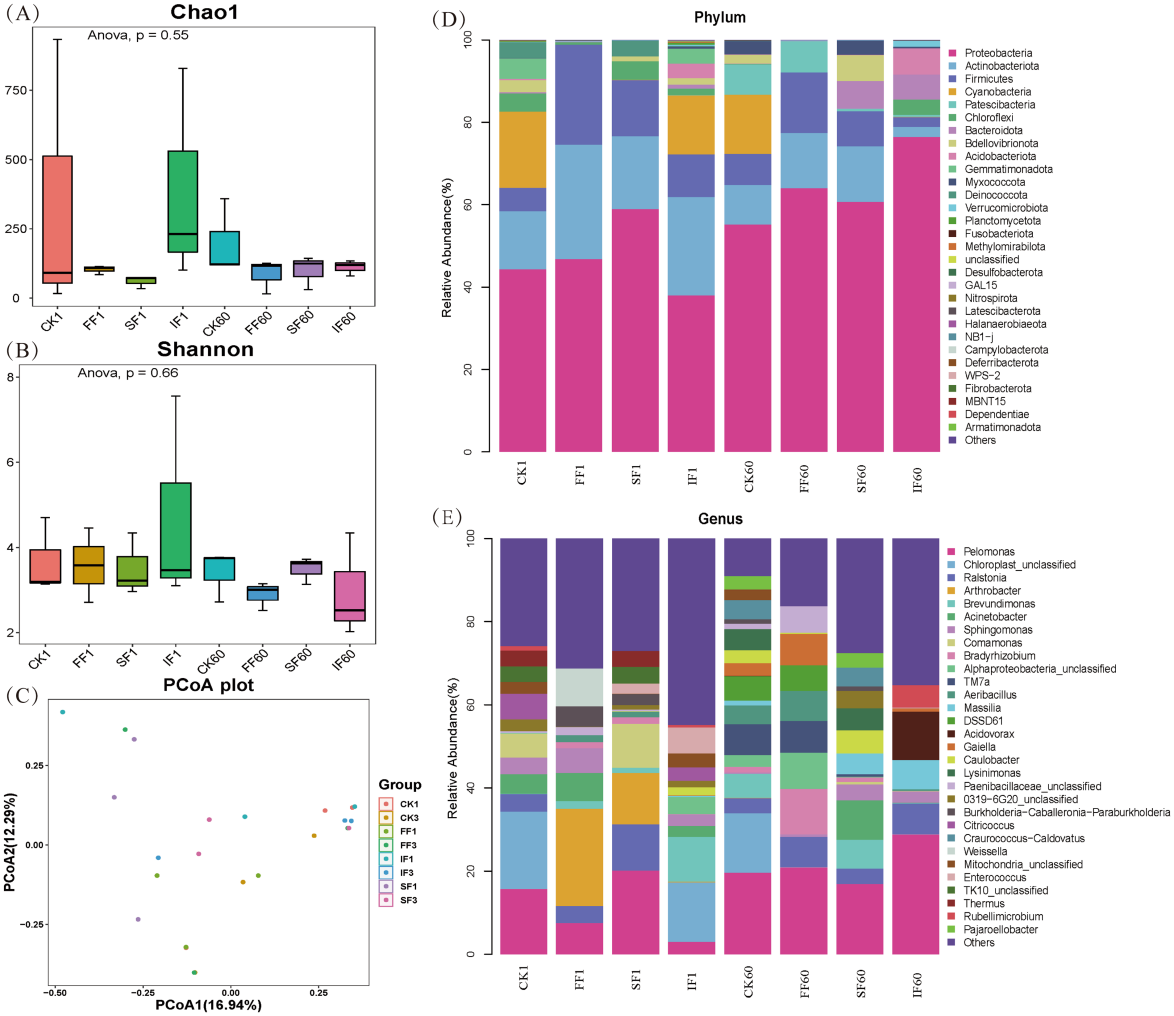
Analysis of bacterial community **(A)** Chao1 index, **(B)** Shannon index, and **(C)** PCoA in rhizosphere soil sample from different fertilization treatments, as well as the relative abundance of bacterial community at the **(D)** phylum level, and **(E)** genus level.

To analyze the rhizosphere microbial response mechanism of using FF to remediate antimony-contaminated soil, we performed bacterial 16S rDNA sequencing of the rhizosphere soil sample of *P. vittate* on day 1 and day 60. Then, the microbial diversity and community composition differences among different treatments were analyzed. A total of 24 samples from the CK, FF, SF, and IF groups were sequenced, double-ended spliced, quality controlled, and chimeric filtered to obtain 2,240 ASV. Then, based on the ASV abundance table, microbial *α*-diversity and *β*-diversity analyses and taxonomic identification at the phylum and genus levels, were performed, and the results are shown in Figure 5.

The Chao1 and Shannon indices were used to evaluate the richness and diversity of the microbial community in the samples, respectively. Applying FF and SF resulted in a slight reduction in the Chao1 index but no significant change in the Shannon index (Figures 5A,B). There were no significant variations in Chao1 and Shannon indices among the four treatments on day 1 or after 60 days of fertilizer application (*p* > 0.05). It is worth mentioning that after 60 days of fertilization, the Chao1 and Shannon indices of the FF group were lower than those of the control group. This may be due to the application of FF-enriched certain particular microbes in the rhizosphere soil of *P. vittate*, reducing community richness and diversity. The PCoA analysis results showed that there was a considerable degree of dispersion among the samples of the four treatments on the first day of fertilization (Figure 5C). After 60 days, some samples in the organic fertilizer group and IF group were relatively compact. Still, all of them were dispersed at a distance from the samples in the control group. This finding suggested significant differences in the structure of the rhizosphere soil microbial community among the four treatments on the first day of application. However, after 60 days, there was a higher degree of similarity in the community structure in the three groups of fertilizer application, compared with the control group.

To investigate whether the application of FF could enrich certain specific microbes in the rhizosphere soil of *P. vittate*, samples from different fertilization treatments were classified and identified at the phylum and genus levels. Bacteria with relative abundance above 5% at the phylum level in all samples were *Proteobacteria*, *Actinobacteria*, *Firmicutes*, *Cyanobacteria*, *Patescibacteria*, *Chloroflexi*, *Bacteroideta*, *Bdellovibrio*, and *Acidobacteria*. Currently, nearly a hundred bacteria have been reported to possess the ability to oxidize Sb(III), mainly from four phyla: *Proteobacteria*, *Actinobacteria*, *Firmicutes*, and *Bacteroidetes* (Xiao et al., 2023). The relative abundance of these four phyla reached 99.11% in the FF group and 90.33% in the SF group on day 1 of fertilization, while it was only 73.21 and 64.49% in the IF and control groups, respectively. Over time, the microbial community structure of rhizosphere soil underwent succession. However, *Proteobacteria*, *Actinobacteria*, *Firmicutes*, and *Bacteroidetes* still maintained a dominant position in the four groups, with total relative abundances of 92.12, 89.41, 87.43, and 72.49% in the FF, SF, IF, and CK groups, respectively. This demonstrated that applying FF helps to increase the population of antimony oxidizing bacteria in antimony-contaminated soil and can be continuously enriched to the endosphere of *P. vittate*.

We further identified seven bacterial taxa with Sb(III) oxidation function in the genus level analysis: *Acinetobacter*, *Sphingomonas*, *Comamonas*, *Bradyrhizobium*, *Alphaproteobacteria*, *Acidovorax*, and *Paenibacillaceae_unclassified* (Deng et al., 2021). The total relative abundance of these seven bacterial taxa in CK1, FF1, SF1, IF1, CK60, FF60, SF60, and IF60 was 14.92, 16.19, 12.46, 9.59, 5.79, 26.55, 15.24, and 14.6%, respectively, indicating that the relative abundance of Sb(III) oxidizing bacteria in the FF group was consistently higher than that in other groups throughout the entire study period. Shi et al. (2013) isolated 36 strains of Sb(III) oxidizing bacteria, mainly *Acinetobacter*, *Comamonas*, and *Pseudomonas*, from multiple mining areas samples in China. Yu et al. (2021) screened a bacterial strain with arsenic-antimony oxidizing ability in mining soil. They identified as *Bradyrhizobium*, which grew stably on the root of *Vetiver zizanioides*. *Alphaproteobacteria* was recognized as the predominant bacteria in the root samples of *P. vittate* from lead-zinc mining areas, possessing the arsenic antimony oxidation gene *aoxB* (Sun, 2019). Furthermore, *Bradyrhizobium* and *Paenibacillaceae* possess excellent symbiotic nitrogen fixation and phosphorus and potassium solubilization-promoting functions and are commonly used as biofertilizers in agricultural production (Liu, 2014). The relative abundance of *Bradyrhizobium* and *Paenibacillaceae* in the rhizosphere soil of the FF group was significantly higher than the other three groups (*p* < 0.05). This finding indicated that the use of FF helps nitrogen-fixing, phosphorus-solubilizing, and potassium-solubilizing microorganisms to colonize at the root of *P. vittate*, thereby promoting the accumulation of NPK nutrients to cope with the poor soil environment of mineral mountains.

## Conclusion

After applying FF, SF, and IF to the antimony-contaminated soil, the soil physicochemical properties were improved to varying degrees, and the application of FF had the most significant effect in reducing bulk density, improving soil salinization, increasing soil OM and available NPK content (*p* < 0.05). FF could promote the oxidation and detoxification of Sb(III) in the soil to produce Sb(V), which is further enriched to the endosphere of *P. vittate*. Microbial community analysis showed that the application of FF promoted the continuous enrichment of *Proteobacteria*, *Actinobacteria*, *Firmicutes*, and *Bacteroidetes* in the root of *P. vittate*, especially the specific microbial groups with Sb(III) oxidation, nitrogen fixation, and phosphorus and potassium solubilization functions such as *Acinetobacter*, *Sphingomonas*, *Comamonas*, *Bradyrhizobium*, *Alphaproteobacteria*, *Acidovorax*, and *Paenibacillaceae*, which can alleviate the stress of poor soil conditions and heavy metals on the growth of *P. vittate* in mines. This study elucidated the rhizosphere microbial response mechanism of using FF to remediate antimony-contaminated soil and provided a new approach for the resource utilization of food waste and the remediation of antimony-contaminated sites.

## Data Availability

The datasets presented in this study can be found in online repositories. The names of the repository/repositories and accession number(s) can be found in the article/supplementary material.
